# Detecting the Effects of the Glucocorticoid Dexamethasone on Primary Human Skeletal Muscle Cells—Differences to the Murine Cell Line

**DOI:** 10.3390/ijms21072497

**Published:** 2020-04-03

**Authors:** Eva K. Langendorf, Pol M. Rommens, Philipp Drees, Stefan G. Mattyasovszky, Ulrike Ritz

**Affiliations:** Department of Orthopedics and Traumatology, University Medical Center of the Johannes Gutenberg University Mainz, 55131 Mainz, Germany; eva.langendorf@unimedizin-mainz.de (E.K.L.); pol.rommens@unimedizin-mainz.de (P.M.R.); philipp.drees@unimedizin-mainz.de (P.D.); stefan.mattyasovszky@unimedizin-mainz.de (S.G.M.)

**Keywords:** atrophy, MuRF-1, myotubes, myoblasts, MAFbx, glucocorticoids, dexamethasone, Foxo, Myosin

## Abstract

Skeletal muscle atrophy is characterized by a decrease in muscle fiber size as a result of a decreased protein synthesis, which leads to degradation of contractile muscle fibers. It can occur after denervation and immobilization, and glucocorticoids (GCs) may also increase protein breakdown contributing to the loss of muscle mass and myofibrillar proteins. GCs are already used in vitro to induce atrophic conditions, but until now no studies with primary human skeletal muscle existed. Therefore, this study deals with the effects of the GC dexamethasone (dex) on primary human myoblasts and myotubes. After incubation with 1, 10, and 100 µM dex for 48 and 72 h, gene and protein expression analyses were performed by qPCR and Western blot. *Foxo*, *MuRF-1*, and *MAFbx* were significantly upregulated by dex, and there was increased gene expression of myogenic markers. However, prolonged incubation periods demonstrated no Myosin protein degradation, but an increase of MuRF-1 expression. In conclusion, applying dex did not only differently affect primary human myoblasts and myotubes, as differences were also observed when compared to murine cells. Based on our findings, studies using cell lines or animal cells should be interpreted with caution as signaling transduction and functional behavior might differ in diverse species.

## 1. Introduction

Lower back pain is becoming more and more common in society and is often associated with skeletal muscle atrophy. Patients with non-specific back pain frequently end up physically restricted with functional disorders and injuries in the back [1,2,3]. Another reason for lower back pain is muscle atrophy caused by denervation, immobilization, malnutrition, the natural aging processes, illnesses, physical inactivity, or genetic factors [4].

Atrophy in skeletal muscles is characterized by a decrease in muscle fiber size [5]. The cell and protein synthesis of skeletal muscle is regulated by complex signaling pathways [6]. The steady state in muscle tissue depends on the balance between anabolic and catabolic metabolism reactions, which ensure a constant level of essential proteins and nutrition factors. A decrease in protein synthesis or an increase in proteolysis may cause an imbalance [7] that can be initiated by molecular triggers leading to the disruption of signal transduction cascades. A reduction of protein synthesis leads to a rapid degradation of contractile muscle fibers [8].

As a result of the lower synthesis rate structural proteins, muscle structure and finally the function cannot be maintained [9]. Glucocorticoids (GC) belong to the factors that may decrease the rate of protein synthesis and increase protein breakdown resulting in muscle atrophy [10,11,12]. It has been demonstrated that denervation and GC treatment induce protein degradation resulting in a loss of myofibrillar proteins and muscle mass [13]. This effect is supported by the fact that GCs increase the proteasome-dependent protein degradation [14] and inhibit protein synthesis [15].

GCs are endogenous stress hormones and are clinically used as anti-inflammatory and immunosuppressive agents [16]. The effect of GCs is mediated by the glucocorticoid receptor (GR), which is located in the cytoplasm. GCs enter the cell and bind to a ligand binding domain (LBD) of the GR. This binding leads to a conformational change and its nuclear translocation. There, GR binds to glucocorticoid response elements (GREs) and activates the transcription of responsive genes or interacts with negative GREs to suppress the expression of specific genes [17,18]. One target is the *Foxo* (forkhead transcription factor O) promoter, where GREs are located, and after the binding of GR, *Foxo* expression is induced [19].

However, high therapeutic doses and prolonged intake can induce undesired side-effects, including osteoporosis, diabetes, and hypertension [16,20,21]. In muscle tissue GCs can induce atrophy due to their catabolic effects on several tissues [22] and causes muscle weakness [23,24]. 

The intracellular signaling pathway PI3K/Akt was also reported in GC-induced atrophy [15,25]. 

*Foxo* is one of the transcription factors that triggers a signaling cascade and thereby activates the muscle-specific ubiquitin ligases *MAFbx* and *MuRF-1* (*muscle atrophy F-box/atrogin-1 and muscle RING-finger protein-1*). These two E3 ubiquitin ligases are associated with muscle atrophy and are involved in the accelerated breakdown of contractile proteins [26] as they stimulate proteolysis by the ubiquitin proteasome pathway (UPS) [15,27,28,29,30]. Normally *Foxo* is phosphorylated, inactive, and stays in the cytoplasm. But dephosphorylated *Foxo* is transferred to the nucleus, where it induces the expression of its target genes *MuRF-1* and *MAFbx* [31,32,33]. The therapeutical use of GCs, for example dexamethasone (dex), leads to an increased expression of these ligases and results in muscle weakness [15,27,28,34,35]. This induced muscle loss could be ameliorated by using the glucocorticoid receptor antagonist „RU-486“ [36].

Another cause of GCs inducing muscle atrophy might be by inhibiting myogenesis via downregulation of *Myogenin* (*MyoG*), a transcription factor for differentiation of satellite cells [37].

There are numerous studies reporting on muscle atrophy caused by dex in vitro in murine or in rat cells, but there is a lack of studies using primary human muscle cells. Moreover, not only did the experimental design including incubation time and applied concentrations of dexamethasone vary, but also the results are partly contrary. 

### Aim of the Study

In this study, we analyzed for the first time the influence of the synthetic GC dex on muscle atrophy in primary human myotubes. We included the analyses of the gene and protein expression of transcription factors of a signal transduction cascade for the development of muscular atrophy and compared our results with the reported studies in murine and rat myotubes. 

## 2. Results

### 2.1. Immunfluorescence and Flow Cytometer Analysis: Identification of Primary Human Myoblasts 

**Figure 1:**
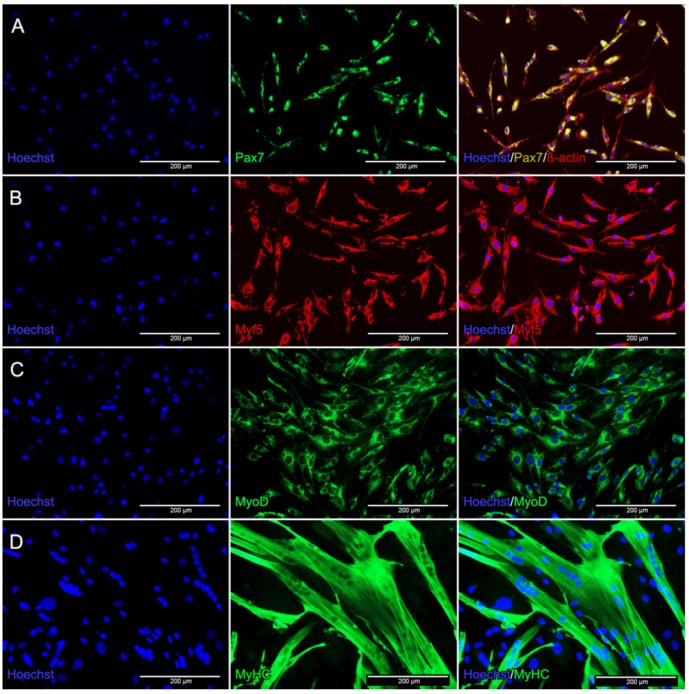
Immunofluorescence staining of primary human myoblasts and multinucleated myotubes. Myoblasts were stained for Pax7 and β-actin (**A**), Myf5 (**B**), and MyoD (**C**). After differentiation, cells were stained for Myosin (**D**). Nuclei were stained with Hoechst dye (blue).

After isolation, cells were characterized as myoblasts by immunofluorescence staining using specific skeletal muscle antibodies for Pax7 (paired box factor 7), Myf5 (myogenic factor 5), and MyoD (myogenic differentiation) (Figure 1A–C). For myotube detection after differentiation, the resulting myotubes were stained with Myosin (MyHC) (Figure 1D). Detection of NCAM (neuronal cell adhesion molecule) has been published previously [38].

Isolated cells express the satellite cell marker Pax7 (A) and are also positive for the myoblast markers Myf5 (B) and MyoD (C). This confirms their myoblastic phenotype. However, the number of Pax7^+^ cells was significantly lower compared to the other myogenic markers, which verified that the isolated cells were myoblasts instead of satellite cells. Differentiated myoblasts stained positive for the myotube marker Myosin and showed the typical multinucleated myotube shape.

#### Flow Cytometer Analysis

Primary human myoblasts were characterized in individual passages (0–4). As these cells are primary cells, they are not used for experiments after passage 4. After this passage, primary cells can change their phenotype and genotype. As a control, IgG APC (Immunoglobulin G Allophycocyanin) coupled cells were used to correct the measured values and to exclude vital and dead cells. Measurements were carried out in triplicates.

**Figure 2:**
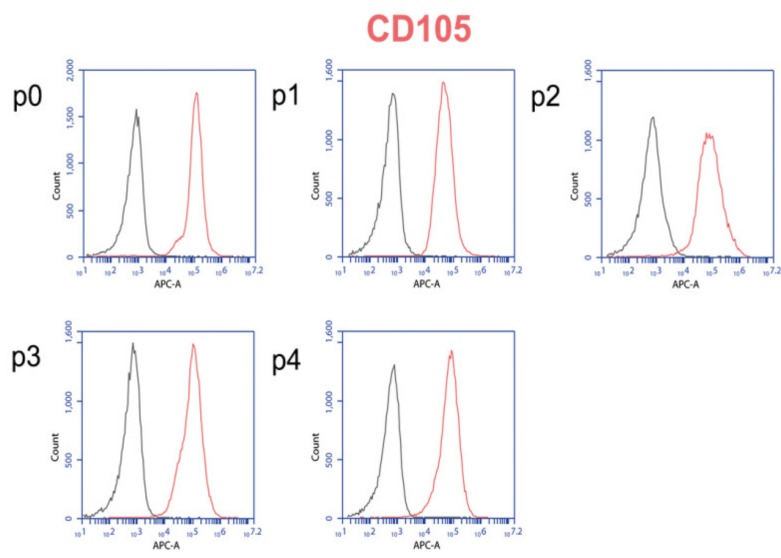
Flow cytometric analysis of the surface antigen CD105 on primary human myoblasts. IgG APC was used as the isotype control (black), and CD105 conjugated APC antibody (red) was used. The cell count is plotted depending on the fluorescence intensity.

Since myoblasts were isolated from tissue, the flow cytometric analysis was selected for a general examination of the cell populations. We chose CD105 (cluster of differentiation 105), to check whether the isolated cells had mesenchymal stem cell character. Figure 2 illustrates that in passage 0 (p0), 99.2% of the isolated cells express CD105. A comparable result was detected in passage 1 with 99.5% positive cells for CD105. In passage 2, the expression decreases to 94.8%, drops to 92.3% in p3, and drops further to 90% in p4.

### 2.2. Dex Had no Impact on Cell Viability

**Figure 3:**
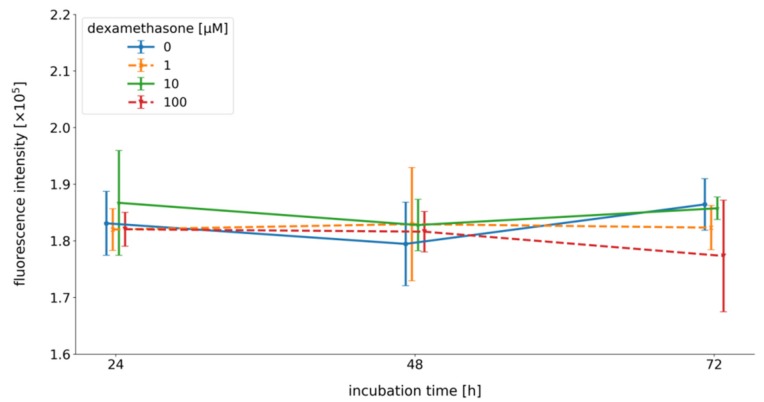
Viability assay of myotubes 24, 48, and 72 h after the incubation with dex. Results are represented in a line diagram and error bars show the standard deviation (*n* = 9).

To analyze the effect of dex on the isolated primary cells, the viability was examined using the AlamarBlue^®^ reagent. After differentiation, cells were incubated with dex with three different concentrations (1, 10, and 100 μM) for a time period of 72 h (Figure 3). 

First, myoblasts were cultured in differentiation medium to induce myotube formation for five days followed by incubation with 1 (orange), 10 (green), and 100 μM (red) dex for 72 h in differentiation medium (DM). Cell viability was measured after 24, 48, and 72 h. Control cells (blue) were incubated without dex. 

The results clearly show that dex had no impact on cell viability compared to untreated control cells (Figure 3).

### 2.3. Gene Expression Analysis of Human Myotubes and Myoblast after Treatment with Dex

The influence of the synthetic GC dex on the gene expressions of primary human myoblasts and myotubes for *MAFbx*, *MuRF-1*, *Foxo*, *Myf5*, *MyoD*, *MyoG*, and *Myosin* was checked via qPCR. Expression levels were compared to untreated cells and evaluated according to the 2^−ΔΔCt^-method. 

#### 2.3.1. Dex Induces the Expression of the Atrophy-Related Genes *MuRF-1* and *MAFbx*

This section is dedicated to the analysis of the mRNA expression after the induction of atrophy in human myotubes with the synthetic GC dex. For this purpose, after differentiation (over 5 days) myotubes were incubated with dex for 48 and 72 h (1, 10, 100 μM each). In addition to gene expression under the influence of dex, the gene expressions of differentiation markers were also examined. The incubation time of 72 h, which we used, has not been published in any study so far. In muscle atrophy, the transcription factor *Foxo* is elevated and activates the upregulation of the E3 ubiquitin ligases *MAFbx* and *MuRF-1*. 

**Figure 4:**
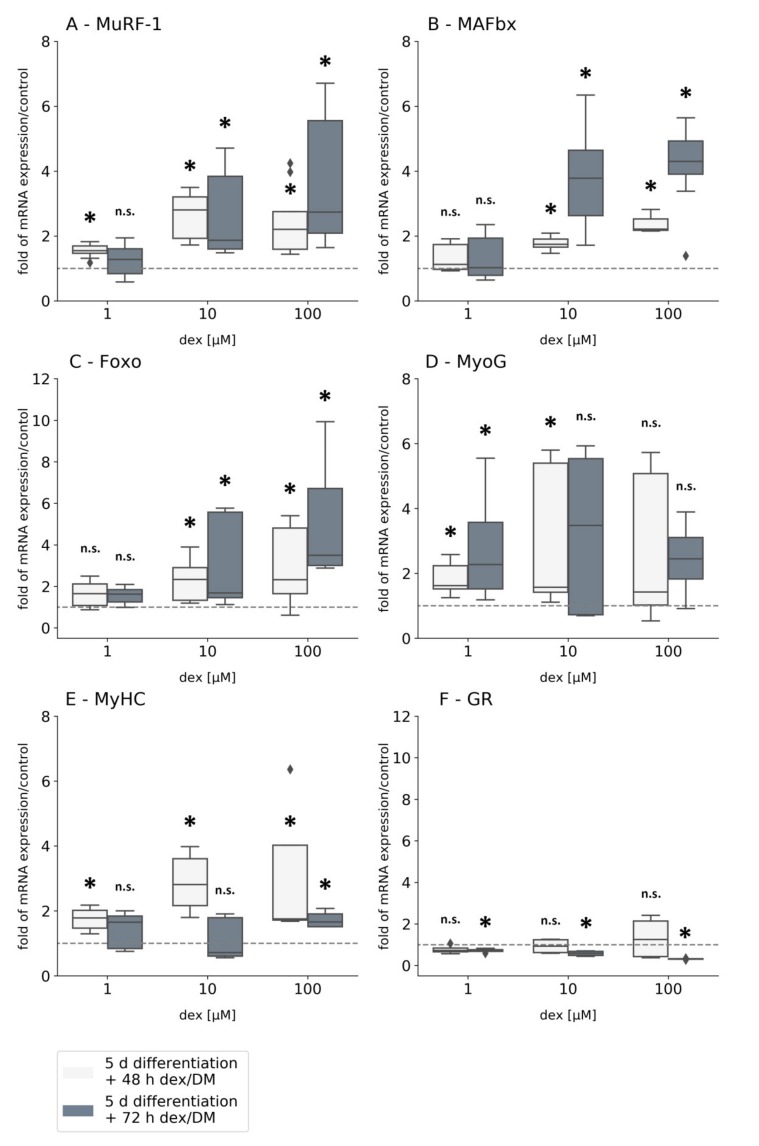
Gene expression analysis of *MuRF-1* (**A**), *MAFbx* (**B**), *Foxo* (**C**), *MyoG* (**D**), *MyHC* (**E**), and *GR* (**F**) in primary human myotubes after dex treatment. The mRNA levels were normalized to *GAPDH* and calculated as ratios in relation to the untreated control group (interrupted line). Experiments were performed in triplicates with myoblasts isolated from three different specimens (*n* = 9). Results are presented as medians and quartiles, and *p* values < 0.05 indicate statistical significance (* *p* < 0.05), and *p* values > 0.05 indicate no statistical significance (n.s. > 0.05). Rhombs are presented as outliers.

The atrophy marker *MuRF-1* was significantly increased after 48 and 72 h at each dex concentration except 1 µM after 72 h compared to the untreated control (Figure 4A). This shows that even low concentrations and incubation times lead to increased mRNA expression of *MuRF-1* by dex. The second ubiquitin ligase *MAFbx* shows significantly increased expression with the concentration of 10 µM after both incubation periods (Figure 4B). Low concentrations of dex have no impact on mRNA of *MAFbx*. The transcription factor *Foxo* is significantly increased expressed after both incubation periods but only using medium and high concentrations (10, 100 µM) of dex; that is similar to the effect on *MAFbx* gene expression (Figure 4C). Gene expression of the myogenic factor *MyoG* is also significantly increased after 48 h using lower concentrations of dex (1, 10 µM), but no significant differences in gene expression were observed after the treatment with 100 µM dex for 48 h compared to control (Figure 4D). A short incubation time of 48 h leads to a significant upregulation of *Myosin* irrespective of the concentration of dex used (Figure 4E). However, after a 72 h incubation period, *Myosin* gene expression was only significantly increased with the highest dex concentration, while medium and low concentrations showed no statistical significant expression changes compared to the untreated control group (Figure 4E). Here dex has a time-dependent effect on the expression of the myogenic differentiation markers *MyoG* and *Myosin*. The expression of the *GR* is not statistically significantly either down or upregulated compared to the untreated control cells at each dex concentration after a 48 h incubation period (Figure 4F). In contrast, its expression was significantly downregulated after cells were incubated for 72 h with 1, 10, and 100 µM dex compared to the untreated control group (Figure 4F). 

#### 2.3.2. Dex has Developmental-Stage-Dependent Effects on Myotubes and Myoblasts

**Figure 5:**
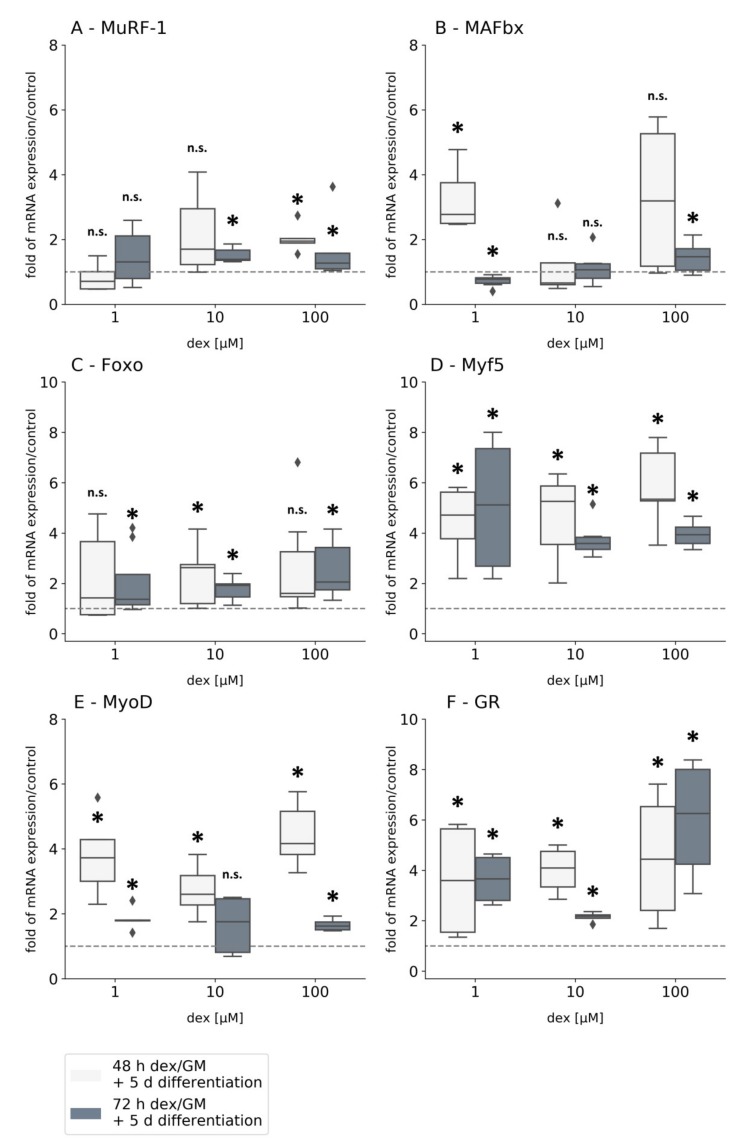
Gene expression analysis of *MuRF-1* (**A**), *MAFbx* (**B**), *Foxo* (**C**), *Myf5* (**D**), *MyoD* (**E**), and GR (**F**) in primary human myoblasts after dex treatment. The mRNA levels were normalized to GAPDH and calculated as ratios in relation to the untreated control group (interrupted line). Experiments were performed in triplicates with myoblasts isolated from three different specimens (*n* = 9). Results are presented as medians and quartiles, and *p* values < 0.05 indicate statistical significance (* *p* < 0.05), and *p* values > 0.05 indicate no statistical significance (n.s. > 0.05). Rhombs are presented as outliers.

The influence of dex should be investigated not only on differentiated myotubes (Figure 4) but also on myoblasts in the proliferating state and their ensuing differentiation into myotubes without dex (Figure 5). For this purpose, cells were incubated for 48 and 72 h with dex in their growth medium (GM) and then differentiated for 5 d without dex.

The treatment of proliferating myoblasts with dex following differentiation without dex led to a significant upregulation of *MuRF-1* after 72 h treatment using medium and high concentrations of dex. Short incubation times of 48 h increased *MuRF-1*’s mRNA level only at a concentration of 100 µM compared to the untreated control (Figure 5A). In contrast, incubation with low concentrations did not result in statistically significant differences. The medium concentrations of dex (10 µM) had no impact on *MAFbx* gene expression after 48 and 72 h (Figure 5B). However, on the one hand 1 µM of dex caused a significant upregulation of *MAFbx* after 48 h, and on the other hand a 24 h longer incubation period caused a significant downregulation of *MAFbx* compared to control cells. High concentrations of dex (100 µM) lead to a significant upregulation of *MAFbx* mRNA after 72 h, but after 48 h *MAFbx* seems to be upregulated in treated cells, although not statistically significantly. 

The results of the gene expression of *MuRF-1* and *MAFbx* at the proliferation state of myoblasts compared to mRNA expression in myotubes indicate that dex has a different impacts on cells depending on the differentiation state (Figure 4A, B). 

After a long incubation time, the transcription factor *Foxo* is upregulated at all concentrations of dex tested. After a 48 h incubation time *Foxo* is only significantly upregulated using 10 µM of dex compared to the untreated control group (Figure 5C). This indicates that dex influences *Foxo* rather after a longer incubation time. However, looking at the myogenic markers, *Myf5* mRNA is significantly upregulated with low, medium, and high concentrations of dex as well as after short and long incubation times (48 and 72 h) compared to the untreated control cells (Figure 5D). These results suggest that the GC dex leads to an enhanced proliferative effect on myoblasts. A similar effect was observed for *MyoD* gene expression (Figure 5E). No statistical significance was observed after 72 h or at a concentration of 10 µM dex compared to the control. The *GR* mRNA expression was significantly upregulated as well, after 48 and 72 h, at low, medium, and high concentrations of dex compared to the untreated control cells (Figure 5F).

Surprisingly, we could not detect gene expression levels of the myogenic markers *MyoG* and *Myosin* in this experiment, suggesting that dex influences the differentiation process in primary human myoblasts. 

### 2.4. Dex has Time and Concentration-Dependent Effects on Myosin Protein Expression in Human Myotubes 

In addition to transcription, the translations of the atrophy-related protein MuRF-1, the atrophy induced transcription factor Foxo, and the muscle protein Myosin in dex-treated human myotubes, were analyzed by Western blot. As described in the literature, MuRF-1 is activated by Foxo, which is followed by binding to Myosin for degradation. Therefore, we focused on the protein expressions of these three proteins. Myotubes were treated after 5 d of differentiation with 1, 10, and 100 µM dex for 48 and 72 h. 

**Figure 6:**
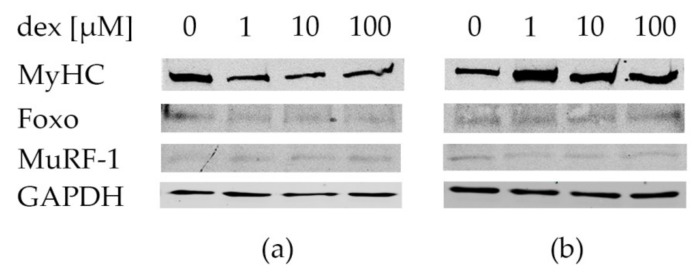
Western blot of differentiated myotubes after dex treatment. Myotubes were incubated with 1, 10, and 100 µM dex for 48 (**a**) and 72 h (**b**). Control cells were incubated without dex (0) and Myosin (MyHC), Foxo, and MuRF-1 protein expressions were detected. GAPDH was used as the loading control.

Protein expression results of myotubes incubated with or without dex for 48 h are shown in Figure 6a. The expression of Myosin is lower in the treated cells compared to control cells without dex. The protein expression of the transcription factor Foxo is very low, both in the control and treated cells, independent of the dex concentrations after an incubation time of 48 h (Figure 6a). MuRF-1 protein is expressed after treatment with 1, 10, and 100 µM dex after 48 h. Its expression was also observed in the untreated control cells, to a lower extent, however, than in the treated cells (Figure 6a). As shown in Figure 6b, Myosin protein expression is higher after the incubation with 1, 10, and 100 µM dex compared to the untreated control cells, and the highest expression was observed after an 72 h incubation period using 1 µM. The protein expression of Foxo was also observed in the control and in the treated cells after 72 h and with no qualitative differences (Figure 6b). The expression of the ubiquitin-ligase MuRF-1 was also expressed in the control and in treated myotubes (concentration independent) after 72 h. 

**Figure 7:**
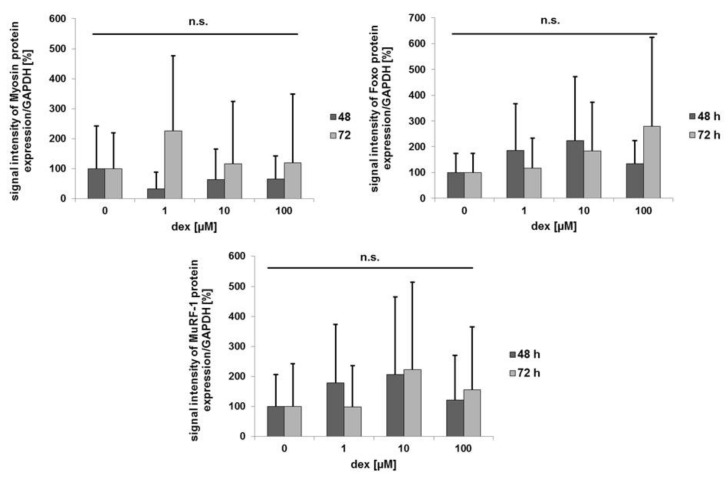
Densitometrical analysis of protein expression. The signal intensities of Myosin, Foxo, and MuRF-1 were normalized to the loading control GAPDH. Measurements were performed in duplicates and repeated three times, and error bars show the standard deviation (*n* = 6). Data were normalized to a control of 100%, and *p* values > 0.05 indicate no statistical significance (n.s. > 0.05).

For protein expression quantification, the signal intensities of Myosin, Foxo, and MuRF-1 were normalized to the signal intensity of GAPDH (Figure 7). The intensity of the normalized control group was compared to 1, 10, and 100 µM dex after 48 and 72 h. Regarding Myosin protein expression, no statistically significant differences were determined between the treatments with 1, 10, and 100 µM and the control group after 48 or after 72 h. The statistical analysis of Foxo showed no significances between the control group and the treated cells. The same conditions were observed for MuRF-1 protein expression in the comparison. However, signal intensity of Myosin seems to be decreased after the treatment with 1, 10, and 100 µM dex after 48 h. But the opposite effect was observed after the incubation for 72 h. The results showed a higher expression of Myosin after the treatment with 1 µM compared to control, which was also reflected by the protein bands—Figure 6a. Expression intensity of Foxo was increased with 1 and 10 µM but was decreased a little with the highest dex concentration after 48 h. After 72 h its expression was increased with higher dex concentrations compared to the control. After 48 h, the signal intensity of MuRF-1 was increased after the treatment with low and medium dex concentrations. But after the treatment with 100 µM its intensity was little decreased. Similar results of the intensities were observed after 72 h.

## 3. Discussion

### 3.1. Identification of Primary Human Myoblasts

The myoblastic phenotype of the isolated cells was confirmed by the high expression levels of the myoblast specific markers Myf5 and MyoD (Figure 1B–D). As previously reported by other groups, these three proteins are predominantly expressed by proliferating myoblasts [39,40,41].

As expected, we could only detect a low number of Pax7^+^ cells (Figure 1A) [42] as our isolation procedure is specific for myoblasts and not for satellite cells. The enzymatic digestion to dissociate muscle tissue into a single cell suspension and the environment of cell culture in myogenic medium may be a possible explanation for the activation of a low number of satellite cells [43].

Differentiation of myoblasts to myotubes was induced by serum reduction, which is an established standard method [43]. Myosin expression in our primary human cells demonstrated the presence of myoblasts differentiating into multinucleated myotubes. This phenomenon is comparable to studies performed with the murine C2C12 cell line [44].

In a next step, the expression of the mesenchymal stem cell marker CD105 was analyzed in our primary human cells to detect whether stem cells were present in the isolated cell population. Flow cytometric analysis revealed that our primary myoblasts were highly positive for CD105 (99.5%) (Figure 2), indicating that they could differentiate from stem cells [45,46]. 

### 3.2. Dex has no Impact on Cell Viability 

As illustrated in Figure 3, we could not detect any toxic effect of the GC dex on primary human myotubes, irrespective of the used concentrations. This has also been described for other cells [47].

### 3.3. Dex Induces the Expression of the Atrophy-Related Genes MuRF-1 and MAFbx

GCs, such as dex, can suppress the PI3K/Akt/mTOR (mammalian target of rapamycin) signaling pathway, activate muscle-specific ubiquitin-ligases, and induce the proteolysis of muscle proteins [26,36,48]. In our present study, primary human myotubes were incubated for 48 and 72 h with dex, and gene expression analysis was performed. The transcription factor *Foxo* was significantly upregulated in myotubes after incubation with 10 and 100 µM dex after 48 and 72 h (Figure 4C). A possible explanation could be that *Akt* and *mTOR* have not been phosphorylated, which led to their inactivation and to the activation of *Foxo* at higher dex concentrations [49]. Similar results were observed in the murine C2C12 myotubes, indicating that dex had a comparable effect on human and murine myotubes [49]. However, a concentration of 100 µM dex resulted in the highest *Foxo* expression in the human primary myotubes (Figure 4C). In contrast, *Foxo* is significantly upregulated in the murine C2C12 cell line already at 1 μM dex [28]. Although our study demonstrated an upregulation of *Foxo* by dex, an even higher impact could be observed in the murine C2C12 myoblast cell line. Sandri et al. described dex in murine cells as being able to inhibit *Akt* and *mTOR* phosphorylation, leading to a reduction of activity [28,32]. In consequence, it can be assumed that *Foxo* is also dephosphorylated in human myotubes by dex. This is associated with the suppression of Akt and mTOR, and the translocation of *Foxo* into the nucleus [33], where the expression of the E3 ligases *MuRF-1* and *MAFbx* will be induced [50,51,52,53,54].

*MuRF-1* gene expression is significantly increased at low dex concentrations after only a short incubation time (1 µM and 48 h), and it increases even further at higher dex concentrations (Figure 4A). This gives clear evidence that the induction of *MuRF-1* gene expression by GCs can be triggered in human myotubes and is consistent with previously published findings in murine cells [28].

In a direct comparison of rat and murine myotubes, it was demonstrated that dex has a stronger effect on *MuRF-1* expression in rat L6 myotubes (at 50 nM) than on murine C2C12 myotubes compared to the present study using human myotubes using concentrations in the micromolar range. In the mentioned study, they did not observe any significant differences on *MuRF-1* gene expression in the murine C2C12cell line [55]. In summary, there are differences in both species and differences in the results regarding the effect of dex on *MuRF-1* expression. 

*MAFbx* expression was significantly increased in human myotubes. However, in contrast to *MuRF-1* gene expression, this effect was only detectable after incubation with medium and high dex concentrations (10 and 100 µM) (Figure 4B). 

These results argue for the initiation of muscle atrophy and are comparable with reported studies working with murine cells [27,56,57]. In contrast, in the rat L6 myotubes, the *MAFbx* expression was higher at 50 nM dex than at nM [55] showing different effects of dex in different species. The time- and concentration-dependent influence on gene expression of both E3 ligases confirms the induction of atrophy in human myotubes corresponding to reactions in C2C12 [58,59] and rats [26].

*MyoG* is described as a substrate of *MAFbx* [29,60,61]. It has been demonstrated that in the C2C12 cell line after incubation with dex *MyoG* is poly-ubiquitinated and degraded, leading to an inhibition of the differentiation from myoblasts to myotubes [62,63]. In our experiments with human myotubes, the mRNA expression of *MyoG* was significantly upregulated by 10 µM dex (Figure 4D). In conclusion, dex had a different effect on *MyoG* gene expression in human myotubes compared to murine cells. Although both studies observed increased MAFbx expression, dex did not affect *MyoG* gene expression in human myotubes. These results suggest a species-specific influence on *MAFbx* and *MyoG* gene expression. 

The mRNA level of *Myosin* was elevated in human myotubes by dex (Figure 4E). In the literature, different effects are described concerning the effect of dex in C2C12 cells. One study describes an inhibited *Myosin* expression [57], whereas another study showed an upregulation of myogenic differentiation factors after the treatment with 100 µM dex [64].

The increased *Myosin* expression in human primary myotubes, using low and high concentrations of dex, indicates that dex strengthens the differentiation process, as described for murine cells by Han et al. [57]. *MuRF-1* acts as a general regulator of protein degradation and participates in cell metabolism [65]. Myosin is a target of MuRF-1, whereby the latter binds to Myosin and marks it for proteasomal degradation [28,66]. Our results indicate that dex acts as an enhancer for *Myosin* mRNA expression. Another explanation might be that cells counterregulate a degradation process by inducing *Myosin* expression. It is possible that *MuRF-1* does not affect gene expression but rather influences protein expression either posttranscriptionally or posttranslationally. Stitt et al. described a dose dependent expression of *MAFbx* by dex after 24 h. The same effect was seen in human myotubes [59]. However, *MAFbx* expression showed no significant increase in human myotubes using 1 µM compared to untreated control cells. Surprisingly the *GR* was either significantly down (72 h) or not statistically significantly expressed at each dex concentration when compared to the untreated control cells (Figure 4F). Similar results were found in the rat L6 and the mouse C2C12 cell lines [55]. It was demonstrated that the mRNA expression was either significantly downregulated in C2C12, or, in rat L6 myotubes, no expression changes of the *GR* could be observed. 

In summary, it can be concluded that dex has different regulating effects on gene expression in murine, rat, and human myotubes. 

### 3.4. Dex has Developmental-Stage-Dependent Effects on Myotubes and Myoblasts

The presented results (Figure 5) showed that dex has an inhibiting effect on the differentiation from human myoblasts to myotubes. This is demonstrated by the lacking expression of the myogenic terminal differentiation genes *MyoG* and *Myosin* with the very high expressions of the proliferation genes *Myf5* and *MyoD* (Figure 5D,E). 

In human myoblasts a clear proliferation-enhancing effect can be observed by dex, whereas at the same moment the differentiation is inhibited. Both E3 ligases are expressed, but dex has a lower effect on *MuRF-1* expression, as it is only expressed at high concentrations and after long incubation times compared to the untreated control group (Figure 5A). These results indicate that the impact of dex on *MuRF-1* gene expression is lower in human myoblasts than in human myotubes (Figure 4A). Consequently, the activation of proteolysis by *MuRF-1* is limited. Furthermore, the expression of *MAFbx* was less influenced by dex compared to the untreated control. Depending on the duration and concentration of dex incubation, it is differently expressed. (Figure 5B). According to our results, dex can activate the expression of both ligases without having a profound effect on proteolysis or muscle genes.

The elevated time-dependent expression of the transcription factor *Foxo* in our study (Figure 5C) clearly indicates the induction of atrophy in human myoblasts in vitro. There was no association between *Foxo* expression and the expression of *MuRF-1* and *MAFbx*. These results indicate that the effect of dex in human myoblasts is not comparable with human myotubes (Figure 4).

The significantly upregulated gene expression of *Myf5* at low, medium, and high dex concentrations and after 48 and 72 h incubation suggests an increased proliferation rate. This is associated with a suppressed differentiation process, which was determined by the lack of *MyoG* and *Myosin* gene expression. The high level of *Myf5* gene expression indicates that dex inhibits the differentiation process and favors a hypertrophic state of the cells. Comparable effects were detected in murine C2C12 cells [44,67], suggesting a similar dex effect irrespective of the species. The upregulated gene expression of *MyoD* verified that dex enhanced the proliferation rate (Figure 5E).

In the study of Han et al. using murine C2C12 cells, dex enhanced differentiation after the incubation. In contrast to our results (Figure 5) *MAFbx* was repressed while *Myosin* expression was increased [57]. *Foxo* expression clearly indicates an induction of muscle atrophy in murine as well as in human muscle cells in vitro [50,51,52,53,54]. With regard to the supplements in the growth medium of myoblasts, the growth factor bFGF (basic fibroblast growth factor) was added which could also be a reason for delayed differentiation [68]. 

We found a significant upregulation of the *GR* when cells were treated for 48 and 72 h with dex in the proliferative state followed by their differentiation without the GC. The group of Sun et al., found that GR protein is abundantly expressed during the differentiation process of the murine C2C12 after dex treatment [69]. The results in our present study show that the *GR* is constantly expressed in our cells at the mRNA level. That constant expression and the fact that the cells showed no expression of the terminal differentiation markers *MyoG* and *Myosin* (Figure 5) could indicate that the *GR* is only significantly expressed before cells undergo their terminal differentiation. We hypothesize that the constant expression of the *GR* in response to dex could have resulted either from the beginning or from the unreached terminal differentiation stage related to the expressions of *MyoG* and *Myosin*. This could also be associated with the repressed expression of the *GR* in the differentiated myotubes after dex treatment (Figure 4F), suggesting that *GR* expression after dex treatment depends on the developmental or the differentiation stage in human cells. A study found that glucocorticoid treatment reduces the differentiation of murine C2C12 myoblast and suggests that these results are caused by the reduction of the differentiation-specific *MyoG* mRNA level [37].

### 3.5. Dex has Time and Concentration Dependent Effects on Myosin Protein Expression in Human Myotubes

As was already shown in studies using murine C2C12 myotubes, GCs are able to induce atrophic conditions, and therefore atrogenes will be activated, leading to protein degradation [22,55]. In particular it has been demonstrated that dex can induce protein degradation by upregulating genes of the ubiquitin-proteasome signaling pathway in vitro [56,59].

In the present in vitro study, it could be observed that in primary human myotubes, treatment with 1, 10, and 100 µM dex for 48 h lead to decreased Myosin protein expression (Figure 6a). This indicates that dex had the same impact on the Myosin protein expression after differentiation in human myotubes (five days of differentiation and 48 h dex), as described from murine myotubes [30,66]. That decrease in Myosin protein expression after dex treatment was confirmed in the quantification of the signal intensity compared to GAPDH (Figure 7). Despite the shown data, the differences between the groups are not statistical significant, which could be caused by the high standard deviations. These high deviations could result from the three myotube cultures from three different donors which were used to perform the experiments. The comparison between the control and the treated groups showed that Myosin protein expression is decreased after 48 h incubation with dex (Figure 7). The opposite effect was observed after 72 h. The dex treatment increased the Myosin protein expression compared to the untreated control group. These results could also be observed in the quantifications. Interestingly, these results are the opposites of the results obtained in murine myotubes [30,66], again confirming species-specific differences. Moreover, these results confirm gene expression analysis (Figure 4E), where Myosin is also higher expressed in the treated cells than in the control group. 

Regarding Foxo protein expression, it is also expressed after the treatment with dex after 48 h and 72 h in the unphosporylated state but also in the control group (Figure 6a,b), indicating that its expression is independent of dex. This result was also shown in murine C2C12 myotubes after dex treatment and starvation [28]. However, we could observe that the signal intensity of Foxo protein increased after 72 h with higher concentrations of dex (Figure 7). This observation indicates that the effects of dex are similar in human and murine myotubes. In the line with this, MuRF-1 expression could also be detected after differentiation and 48 and 72 h dex incubation in each treated group in our human primary myotubes, which is the same effect observed in studies of murine C2C12 myotubes [35]. There, a higher MuRF-1 expression combined with a reduction in the Myosin expression using dex was observed, and the quantification of the signal intensity showed the same results in MuRF-1 protein expression as in our human myotubes (Figure 6a, Figure 7). Similar results were detected after a longer incubation period (72 h) (Figure 6b). However MuRF-1 was also expressed and detected in the control groups at each incubation time. Similar effects were also determined in C2C12 myotubes [49]. 

### 3.6. Conclusion

Taking all results into account, dex has different effects on primary human myoblasts and myotubes in terms of the mRNA expression of MAFbx and MuRF-1, as the impact on myoblasts was lower than in myotubes. Myoblasts and myotubes treated with dex showed an enhanced mRNA expression in the myogenic proliferation and differentiation markers, but myoblasts were restricted in their differentiation potential. Some expression analyses, for example, Myosin expression, revealed some differences in primary human, murine C2C12, and rat myotubes, when they were treated with dex, indicating species-specific differences. Our results indicate that results from in vitro studies with rat and murine skeletal muscle cells cannot be transferred to humans. Therefore, we recommend performing more human cell experiments in the future.

#### Summary

In the published articles about in vitro muscle atrophy performed with rat L6 and murine C2C12 myotubes, the treatment with dex led to increased MuRF-1, MAFbx, and Foxo gene and protein expressions. The expression of Myosin protein decreased after dex treatment in these cell lines. In the present study with primary human myotubes, we found out that treatment with dex in the myotube stage leads to significant upregulation of MuRF-1, MAFbx, and Foxo gene expressions compared to the untreated control cells. In our protein analysis, we demonstrated that MuRF-1 and Foxo were also expressed in the control group. Moreover, we detected decreased Myosin protein expression after a 48 h treatment with dex in primary human myotubes but observed the opposite effect after a 72 h treatment with dex in primary human myotubes.

### 3.7. Limitations of the Study 

The present study has potential limitations that have to be considered. In our Western blot analyses, the expression of phosphorylated Foxo, which shows whether it is transcriptionally inactive, was not analyzed. We only focused on the last components of the IGF-I (insulin-like growth factor 1) pathway and did not investigate the NFκB signaling or the autophagy system. We did not implement a dex inhibitor in the gene expression analysis and only analyzed dex as synthetic GC. 

## 4. Materials and Methods 

### 4.1. Cell Culture

#### Isolation of Human Primary Myoblasts

The isolation was carried out according to an established and modified method under sterile conditions [70]. Cells were seeded in collagen coated culture flasks (Collagen Type I, CORNING^®^, Discovery Labware, Amsterdam, Netherlands). 

All patients provided written consent. The study was conducted in accordance with the Declaration of Helsinki. Human skeletal muscle specimens were obtained from lumbar spine during surgery and the use of residual materials was approved by the ethics committee of the Landesärztekammer Rheinland-Pfalz in agreement with the university clinic. Cell media are listed in Table 1. Samples were cut into small pieces (1 mm^2^) followed by collagenase type II (470 U/DMEM-F-12, Worthington Biochemical Corporation, Lakewood, CO, USA; Gibco^®^, Life Technologies, Grand Island, NY, USA) treatment for one hour (37 °C in a water bath) under continuous stirring. To obtain a single cell suspension, we used an additional incubation step by treating samples for 20 min with Trypsin/EDTA (0.025%/0.02%, Biochrom GmbH, Berlin, Germany) as previously described after centrifugation (1600 rpm, 7 min). Cell suspensions were filtered through a 70 µm cell strainer with growth medium (GM), and after centrifugation (1400 rpm, 5 min) cell suspensions were seeded in uncoated cell culture flasks for 2h to obtain a pure myoblasts culture. The supernatant was seeded in collagen coated culture flasks and incubated at 37 °C and 5% CO_2_. Medium was changed every second day. Differentiation of primary human myoblasts to multinucleated myotubes started at a cell density of 70% by switching GM to differentiation medium (DM). Cells were differentiated for 5 d to obtain multinucleated myotubes. 

### 4.2. Immunofluorescence

Identification of myoblasts and myotubes was performed by immunofluorescence staining. Cells were stained with specific antibodies: either Pax7, Myf5, and MyoD for myoblast detection, or Myosin for myotube identification. 

After two washing steps with PBS (Gibco^®^Invitrogen™ Life Technologies, Carlsbad, CA, USA), cells were fixed and permeabilized with ice cold methanol for 20 min followed by several washing steps in PBS. Pax7 (ab199010, 1:200), Myf5 (M-18: sc-31949, 1:100), MyoD (M-318: sc-760, 1:150), and skeletal muscle Myosin (F59: sc-32732, 1:200, Santa Cruz Biotechnology, Dallas, TX, USA) were used as primary antibodies and incubated over night at 4 °C. After washing with 0.5% BSA/PBS cells were stained with Alexa Fluor^®^488 (A11001 and A11008, 1:200, Invitrogen™ Life Technologies, Carlsbad, CA, USA), (Cy™3-conjugated donkey anti-Goat IgG (H+L) 705-165-003, 1:450, Jackson Immuno Research Laboratories, Inc, West Grove, PA, USA) (1h) in the dark at room temperature. After nuclei staining (15 min) with Hoechst 334,565, detection was performed using the EVOS^®^ Digital Inverted Microscope (Life Technologies, Carlsbad, CA, USA).

Experiments were performed 3 times using different myoblasts cells to confirm the results. 

### 4.3. Flow Cytometer Analysis

For surface protein detection, primary human myoblasts (3 × 10^5^) were washed in buffer (PBS, 0.5% FCS, 0.1M EDTA, pH 7.3) and incubated with APC fluorescent conjugated antibodies. Measurements were performed in triplicates for each passage and performed at 488 and 640 nm using the flow cytometer (C6 Flow cytometer, C. Sampler^®^ Accuri Cytometers Inc., Ann Arbor, MI, USA). For a negative control, cells were incubated with IgG APC. The IgG APC coupled cells were used to correct the measured values and to exclude live and dead cells. Measurements were carried out in triplicates at each passage and performed with 3 different myoblast cells isolated from 3 different muscle specimens.

### 4.4. Viability Assay

Dex was used in this study to induce atrophic conditions. First, 10,000 cells were counted and seeded in collagen-coated 24 well plates, and cell viability was analyzed using the AlamarBlue^®^ cell viability reagent (Gibco^®^ Invitrogen™ Life Technologies, Carlsbad, CA, USA) according to the manufacture’s instructions. Measurements were performed in triplicates with the GloMax^®^Multidetection System (Promega, Madison, WI, USA) (extinction 525 nm; emission 580–649 nm), and experiments were repeated 3 times. To test if dex has toxic effects on primary human myotubes, cells were differentiated for 5 days to form their multinucleated myotubes following incubation with 1, 10, or 100 µM dex for 24, 48, or 72 h. Control cells were incubated without dex.

### 4.5. Induction of Atrophic Conditions 

To induce atrophic condition with dex, 50,000 cells/well were seeded in collagen-coated plates. To determine the influence of dex (Sigma-Aldrich^®^GmbH, St. Louis, MO, USA) on myoblasts and the ensuing differentiation, myoblasts were incubated for 48 and 72 h with dex in GM followed by 5 days differentiation without dex. To test dex’s impact on myotubes, cells were differentiated for 5 days followed incubation with 1, 10, and 100 µM dex for 48 and 72 h. All experiments were repeated 3 times with 3 different skeletal muscle specimens. 

### 4.6. Gene Expression Analyses

#### 4.6.1. RNA Isolation and cDNA Synthesis

Total RNA was isolated with the RNA isolation Kit (peqGold, Total RNA Kit, Peqlab Biotechnology GmbH, Erlangen, Germany) according to manufactures instructions, followed by quantification using UV spectroscopy. 

Total RNA (1 µg) was reverse transcribed into cDNA using dNTPs (4you4 dNTPs Mix (10 mM), BIORON GmbH, Ludwigshafen), Random Primers (Promega, Madison, WI, USA), and MuLV RT (M-MuLV Reverse Transcriptase, M0253S New England Biolabs, Ipswich, MA, USA) according to the manufacturer’s instructions. 

#### 4.6.2. Quantitative Real-Time PCR

For gene expression analyses, cDNA template underwent PCR amplification (40 cycles) using the SYBR Green (PowerUp™ SYBR^®^ green master mix, Applied Biosystems, Foster City, CA, USA) and sequence specific primers (Primer sequences listed in Table 2). *GAPDH* was used to normalize gene expression. Sample amplification was performed with the qTower3 (Jena Analytik, Jena, Germany). An initial activation step at 95 °C for 2 min followed by denaturation and enzyme activation at 95 °C for 15 s and 40 cycles at 60 °C for 15 s for annealing and extension. Results were calculated using the well-established 2^−ΔΔ^Ct method [71]; they presented the expression levels as the ratio of gene expression of untreated cells.

### 4.7. Protein Expression 

#### Western Blot

Cells were harvested and homogenized in RIPA buffer (Sigma-Aldrich^®^ GmbH, St. Louis, MO, USA) containing 50 mM Tris-HCl, pH 7.5, 150 mM NaCl, 1 mM EDTA, 1% (*v/v*) NP-40, 0.1% (*v/v*) sodium dodecyl sulfate and protease, phosphatase inhibitor (Roche, Basel, Switzerland). Protein concentrations were determined by DC™ Protein Assay (Bio-Rad Laboratories, Inc., Hercules, CA, USA) and equal amounts of proteins (25 µg) were separated by 8% and 10% SDS-PAGE and transferred to nitrocellulose membranes. Membranes were incubated with antibodies specific to Myosin (1:200), MuRF-1 (1:200), and Foxo (1:200) (all from Santa Cruz Biotechnology, Dallas, TX, USA), and GAPDH (1:10,000, Acris Antibodies GmbH, Herford, Germany), respectively, and appropriate secondary dye-conjugated antibodies goat anti-mouse IRdye800, IRdye650 (1:10,000; LI-COR^®^, Lincoln, NE, USA) to reveal protein bands for 1 h at room temperature. Membranes were scanned using the Odyssey SA Imaging System (LI-COR^®^, Lincoln, NE, USA). Experiments were performed in duplicates and the signal intensity was normalized to GAPDH.

### 4.8. Statistical Analyses

Statistical analyses were performed using SPSS (IBM^®^ GmbH, Ehningen, Germany). The results are presented as medians and quartiles or as means ± standard deviation. Measurements were carried out in triplicates and experiments were independently repeated three times. 

Normally distributed data were analyzed by one-way ANOVA and pairwise comparisons were conducted using a post hoc test. Non-normally distributed data were evaluated with the Kruskal–Wallis test. For pairwise comparisons, the Mann–Whitney U test was used and *p*-values < 0.05 were considered statistically significant (* *p* < 0.05). Data visualization was performed using Python, 3.7.0 (Python Software Foundation, Wilmington, DE, USA). 

## Figures and Tables

**Table 1:** Myoblast and myotube media.

Growth Medium Myoblasts (GM)	Differentiation Medium (DM)
Dulbecco’s modified Eagle medium (DMEM/F-12)(1:1) + GlutaMAX, Gibco^®^, Life Technologies, Grand Island, NY, USA); 10% FCS (Fetal Calf Serum, Biochrom GmbH, Berlin, Germany); 2.5 ng/mL bFGF (BPS Bioscience, San Diego, CA, USA); 1% Pen. Strep.(Gibco^®^, Life Technologies, Grand Island, NY, USA)	(DMEM/F-12)(1:1) + GlutaMAX, 5% Horse Serum (Biochrom GmbH, Berlin, Germany); 1% Pen. Strep

**Table 2:** Primer pairs for human myoblasts (Eurofins Genomics, Ebersberg, Germany).

Primer	Sequence
*GAPDH* Acc.# M33197	FW cgaccactttgtcaagctca RV aggggagattcagtgtggtg
*Myf5* Acc.# NM_005593.2	FW tgcccgaatgtaacagtcct RV ggaactagaagcccctggag
*MyoD* Acc.# X56677.1	FW ggggctaggttcagctttct RV gctctggcaaagcaactctt
*MyoG* Acc.# NM_002479.5	FW gccagactatccccttcctc RV gaggccgcgttatgataaaa
*Myosin* Acc.# Z38133.1	FW ggcaaaacggaaggagctag RV tcttcctcctcctcagctct
*Foxo* Acc.# NM_001455	FW agccagtctatgcaaaccct RV ccaacccatcagcatccatg
*MAFbx* Acc.# NM_001242463	FW cgtttcactttcaccccagg RV actgcatttctcccctccaa
*MuRF-1* Acc.# NM_032588	FW gagccaccttcctcttgact RV tggtgtccttcttccttccc
*GR* Acc.# AB307716	FW caaatcagcctttcctcggg RV ctggcccttcaaatgttgct

## Abbreviations

MyHC	Myosin heavy chain
IGF-1	Insulin-like growth factor 1
mTOR	Mammalian target of rapamycin
NCAM	Neuronal cell adhesion molecule
GAPDH	Glyceraldehyde 3-phosphate dehydrogenase
GM	Growth medium
DM	Differentiation medium
Pen.Strep.	Penicillin/streptomycin
HS	Horse serum
FCS	Fetal calf serum
bFGF	basic fibroblast growth factor
PI3K	Phosphoinositide 3-kinase
IgG	Immunoglobulin G
APC	Allophycocyanin
